# Ocular fixations and presaccadic potentials to explain pareidolias in Parkinson’s disease

**DOI:** 10.1093/braincomms/fcaa073

**Published:** 2020-06-04

**Authors:** Gajanan S Revankar, Noriaki Hattori, Yuta Kajiyama, Tomohito Nakano, Masahito Mihara, Etsuro Mori, Hideki Mochizuki

**Affiliations:** f1 Department of Neurology, Graduate School of Medicine, Osaka University, Osaka 5650871, Japan; f2 Endowed Research Department of Clinical Neuroengineering, Global Center for Medical Engineering and Informatics, Osaka University, Osaka 5650871, Japan; f3 Department of Behavioral Neurology and Neuropsychiatry, Osaka University, Osaka 5650871, Japan

**Keywords:** Parkinson’s disease, eye-tracking, event-related EEG, pareidolia, visual search

## Abstract

In Parkinson’s disease, a precursor phenomenon to visual hallucinations presents as ‘pareidolias’ which make ambiguous forms appear meaningful. To evoke and detect pareidolias in patients, a noise pareidolia test was recently developed, although its task-dependent mechanisms are yet to be revealed. When subjected to this test, we hypothesized that patients exhibiting pareidolias would show altered top-down influence of visual processing allowing us to demonstrate the influence of pareidolic illusionary behaviour in Parkinson’s disease patients. To that end, we evaluated eye-movement strategies and fixation-related presaccadic activity on scalp EEG when participants performed the test. Twelve healthy controls and 21 Parkinson’s disease patients, evaluated for cognitive, visuo-spatial and executive functions, took a modified computer-based version of the noise pareidolia test in a free-viewing EEG eye-tracking experiment. Eye-tracking metrics (fixation-related durations and counts) documented the eye movement behaviour employed in correct responses (face/noise) and misperceptions (pareidolia/missed) during early and late visual search conditions. Simultaneously, EEG recorded the presaccadic activity in frontal and parietal areas of the brain. Based on the noise pareidolia test scores, we found certain Parkinson’s disease patients exhibited pareidolias whereas others did not. ANOVA on eye-tracking data showed that patients dwelled significantly longer to detect faces and pareidolias which affected both global and local search dynamics depending on their visuo-perceptual status. Presaccadic activity in parietal electrodes for the groups was positive for faces and pareidolias, and negative for noise, though these results depended mainly on saccade size. However, patients sensitive to pareidolias showed a significantly higher presaccadic potential on frontal electrodes independent of saccade sizes, suggesting a stronger frontal activation for pareidolic stimuli. We concluded with the following interpretations (i) the noise pareidolia test specifically characterizes visuo-perceptual inadequacies in patients despite their wide range of cognitive scores, (ii) Parkinson’s disease patients dwell longer to converge attention to pareidolic stimuli due to abnormal saccade generation proportional to their visuo-perceptual deficit during early search, and during late search, due to time-independent alteration of visual attentional network and (iii) patients with pareidolias show increased frontal activation reflecting the allocation of attention to irrelevant targets that express the pareidolic phenomenon. While the disease *per se* alters the visuo-perceptual and oculomotor dynamics, pareidolias occur in Parkinson’s disease due to an abnormal top-down modulation of visual processing that affects visual attention and guidance to ambiguous stimuli.

## Teaser

This work is based on a clinical test that evokes illusions to identify hallucinators in Parkinson’s disease. Revankar *et al.* describe task-based mechanistic interpretations of eye-movement strategies and brain potentials to characterize disease-specific changes in visual processing affected by cognitive and visuo-perceptual impairments in patients that manifest these illusions.

## Graphical Abstract

**Graphical Abstract:**
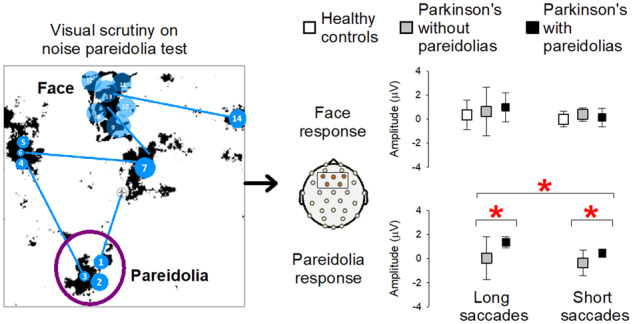

## Introduction

Parkinson’s disease is a debilitating extrapyramidal motor disease, in which patients frequently present with complications of visual hallucinations (Fénelon *et al.*, 2000; Barnes, 2001; Mosimann *et al.*, 2006; Ziemssen and Reichmann, 2007). In these patients, a precursor phenomenon to visual hallucination occurs in the form of misperceptions of meaningful objects or faces arising from ambiguous forms (Uchiyama *et al.*, 2012). These misperceptions are known as ‘pareidolias’, and they are clinically useful in identifying hallucinatory tendencies in such patients (Uchiyama *et al.*, 2012; Mamiya *et al.*, 2016).

Pareidolic illusions are a type of minor hallucinations (Lenka *et al.*, 2019) that are observed at different stages of Parkinson’s disease, involving both cortical (frontal or parieto-occipital cortex) and subcortical structures (upper brainstem or thalamus) (Nishio *et al.*, 2017). Prior studies focusing on hallucinations in Parkinson’s disease have utilized structural, functional or metabolic imaging to demonstrate higher activation of frontal cortices (Oishi *et al.*, 2005; Goetz *et al.*, 2014), posterior cortical grey matter atrophy at the temporo-parietal and medial parietal cortex (Nishio *et al.*, 2017), grey matter atrophy in the medial frontal cortex (Papapetropoulos *et al.*, 2006; Goldman *et al.*, 2014) and in the superior parietal lobe (Ramírez-Ruiz *et al.*, 2008). Interpretation of the results of these studies may be complex (Ffytche *et al.*, 2017), as the approaches used to study hallucinations were indirect and were frequently based on a positive clinical history of hallucinations or lower performance scores on visual perception tests. Whereas these imaging modalities are useful towards understanding minor hallucinations, a mechanistic interpretation via the direct measurement of the cortical activity of patients at the time they are experiencing pareidolias is yet to be clarified.

The functional significance in analysing pareidolias is to understand how subjects perceive a noisy or ambiguous visual stimulus, in which they match sensory information with prior knowledge (Flowers and Robertson, 1995). Pareidolias inherently prompts a visuo-perceptual demand (Uchiyama *et al.*, 2015) that requires reorienting attention based on sensory salience exhibited by the target stimuli. We speculate that the visual information processing system altered in Parkinson’s disease (Stebbins *et al.*, 2004; Li *et al.*, 2010) leads to the interpretation of low-level noisy information as meaningful, owing to an abnormal top-down modulation of the dorsal fronto-parietal goal-driven network (Corbetta *et al.*, 2008; Weil *et al.*, 2016; Nishio *et al.*, 2017). Clinically, pareidolias and hallucinations share common characteristics, and hence the brain responses that are involved in these phenomena are of interest.

To understand the mechanism of the pareidolias occurring in Parkinson’s disease patients, we analysed the patients using the noise pareidolia test (NPT) (Mamiya *et al.*, 2016), which is a test that evokes and quantifies pareidolias, and has been shown to correlate well with the occurrence of visual hallucinations (Uchiyama *et al.*, 2012; Yokoi *et al.*, 2014; Nishio *et al.*, 2017). We evaluated eye-movement strategies and fixation-associated presaccadic activity by scalp EEG using a synchronized EEG eye-tracking system in these patients ‘during’ the NPT. Studies on eye movement metrics evaluating fixation durations and saccade indices have shown how the visual system matches a target encoded in the working memory to guide eye movements towards the target using top-down modulatory control (Hwang *et al.*, 2009; Malcolm and Henderson, 2010). As Parkinson’s disease patients have been reported to have problems with eye fixation as well as with oculomotor or saccadic pursuit of eye movements (Ziemssen and Reichmann, 2007; Matsumoto *et al.*, 2011; Weil *et al.*, 2016), we speculated that changes in eye-tracking are crucial when participants perform the NPT. At a cortical level, the brain generates a slow cortical potential during the presaccadic phase, which reflects the dynamic process of visual encoding, oculomotor construction, and the selection and transfer of attention evolving across time intervals during target identification (Hollingworth, 2004; Gutteling *et al.*, 2010). In a free-viewing visual search task (such as the NPT), presaccadic activity acts as a bridge between the frontal and the parietal cortex, and integrates oculomotor preparation with the covert attentional shift to preferred targets (Nikolaev *et al.*, 2011, 2016). Together with eye-tracking indices, cortical EEG activity enables the analysis of brain-associated processes of stimulus effects, goal-driven selection and the transfer of attention to new targets, and their oculomotor dynamics, features which form the basis of top-down visual processing (Connor *et al.*, 2004; Fischer *et al.*, 2013).

Our main aim in this study was to clarify the top-down modulatory effects causing pareidolias in Parkinson’s disease patients. We hypothesized that if pareidolias were caused by aberrant communication between visual networks, then patients susceptible to pareidolias would show abnormal eye movement characteristics and consequently demonstrate altered presaccadic activity that was distinctly separable from healthy controls and from patients without pareidolic tendencies. From a mechanistic point of view, synchronized EEG eye-tracking of the NPT would offer a more naturalistic approach towards evaluating free-viewing behaviour, which would be suitable to study the phenomenon of pareidolias in such patients.

## Materials and methods

### General information

Twenty-one Parkinson’s disease in-patients [age 70.48 ± 8.54 years (mean, SD)] and 12 healthy controls (age 69.42 ± 8.56 years) were prospectively enrolled for the study. Inclusion criteria for Parkinson’s disease patients were (i) in-patients diagnosed clinically based on Movement Disorder Society Unified Parkinson’s disease rating scale (MDS-UPDRS) (Postuma *et al.*, 2015), (ii) ≥40 years of age, (iii) mini-mental state examination (MMSE) ≥24, (iv) no deep brain stimulation and (v) testing during the ‘on’ state of use of anti-Parkinsonian medications. Parkinson’s disease group were 19 right-handed and 2 left-handed patients, and had obtained a high school certificate or above according to modified Kuppuswamy socioeconomic scale. At the time of testing, most patients were on Levodopa with a combination of other anti-Parkinsonian medication. Only those patients were included who could sufficiently understand the test procedure and have well-defined eye-tracking during the entire experiment. Healthy controls were ≥40 years of age, without any past or present neurological problems, all right-handed with a high school certificate or above. Each participant was tested with a near vision Snellen chart to include normal or corrected-to-normal vision subjects only. Relevant demographic and clinical characteristics of the participants are presented in Table 1. Informed consent was obtained from all subjects with reimbursements provided to healthy controls for the test. The Osaka University institutional review board cleared the protocol for the study to be performed in the Department of Neurology, Osaka University, Japan in accordance with the ethical standards of the Declaration of Helsinki (approval number – 18136).

**Table 1:** Demographic and neuropsychological assessment

Demography		HC (N = 12)	PDnP (N = 11)	PDP (N = 10)	*P*-value	Significance (Dunn’s test)
Age (years)		69.4 (8.5)	65.6 (9.4)	75.8 (1.9)	*0.011*	*PDP > PDnP*
Sex (M:F)		8:4	5:6	5:5		
Disease duration (years)			6.7 (4.0)	9.7 (4.6)	0.133^a^	
LED (mg)			523.4 (360.9)	819.6 (523.0)	0.117^b^	
MDS-UPDRS	Part1		12.3 (7.7)	13.7 (7.2)	0.687^a^	
	Part2		17.3 (9.4)	17.8 (10.5)	0.905^a^	
	Part3		28.6 (13.4)	29.1 (13.4)	0.938^a^	
	Part4		3.6 (4.9)	7.0 (5.6)	0.163^b^	
RBDQ-JP			4.0 (3.3)	5.4 (2.5)	0.342^a^	
**Neuropsychological assessment**						
MMSE			27.0 (2.8)	27.7 (3.0)	0.589^b^	
JART		109.8 (11.2)	105.8 (28.2)	106.8 (10.7)	0.53	
FAB		17.8 (0.4)	15.1 (2.0)	14.8 (1.9)	*<0.001*	*HC > PDP / PDnP*
MoCA		27.0 (1.5)	23.9 (3.8)	21.0 (3.5)	*<0.001*	*HC > PDP*
B-JLO		13.4 (2.1)	11.6 (3.3)	8.67 (4.5)	*0.013*	*HC > PDP*
Modified NPT	Pareidolias	1 (0, 4)	1 (0, 4.7)	24 (16, 42)	*<0.001*	*PDP > HC / PDnP*
	Missed	2 (1.5, 3)	4 (2.2, 6)	4 (1, 6)	0.217	
	D-prime	3.2 (0.7)	2.9 (0.8)	0.4 (0.7)	*< 0.001*	*PDP < HC / PDnP*

Modified NPT scores shown are medians (interquartile range). All other scores are presented as means (standard deviations). D-prime values are adjusted for unequal noise-signal variance.

Three group comparison: Kruskal–Wallis test.

Two group comparison: a Student’s *t*-test, b Mann–Whitney *U*-test.

B-JLO = Benton’s judgment of line orientation test; FAB = frontal assessment battery; HC = healthy controls; JART = Japanese adult reading test scale; LED = levodopa equivalent dose; MDS-UPDRS = Movement Disorder Society—Unified Parkinson’s disease rating scale; MMSE = mini mental state examination; MoCA = Montreal cognitive assessment; NPT = noise pareidolia test; PDnP = Parkinson’s disease non-pareidolia type; PDP = Parkinson’s disease pareidolia type; RBDQ-JP = REM behaviour disorder questionnaire—Japanese.

Significant *P*-values and post-hoc tests are italicized.

### Neuropsychological examination

Neuropsychological tests included—Japanese adult reading test for an estimate of general cognitive status and premorbid intelligence (Bright *et al.*, 2018); frontal assessment battery to evaluate executive function (Dubois *et al.*, 2000); Montreal cognitive assessment to identify mild cognitive impairment (Nasreddine *et al.*, 2005); the short form of Benton’s judgement line orientation test (Form H) for assessment of visuo-spatial function and visual construction (Benton *et al.*, 1978; Gullett *et al.*, 2013); and the original paper-based NPT to evoke pareidolias or visual illusions in Parkinson’s disease patients (Mamiya *et al.*, 2016). Neuropsychological assessments were conducted by a clinical psychologist, independent of experimenters who performed the eye-tracking EEG test.

### Stimuli and experiment details

A total of 80 NPT images (40 images in two consecutive sets) were used as stimuli for testing. Twenty of those images had black and white faces in them. For our study, faces from the original paper-based NPT were replaced by Mooney faces with a shadow-effect that were slightly more ambiguous (Supplementary Fig. 1). We performed this step to (i) avoid the floor-effect with low pareidolia scores on the NPT seen in Parkinson’s disease patients (Yokoi *et al.*, 2014; Nishio *et al.*, 2017), (ii) increase the ambiguity of the images without altering its visuo-spatial characteristics of the face stimuli (Schwiedrzik *et al.*, 2018) from the original NPT. Mooney faces are low-information, two-tone pictures of faces to test face perception—a sorting task in adults which requires configural face processing and is dependent on knowledge-based (top-down) integration of facial features (Cousins *et al.*, 2000; Schwartzman *et al.*, 2008). 2D Mooney face dataset was obtained from the Psychological Image Collection at Stirling webpage made available for public use at pics.stir.ac.uk. The luminance and sharpness of the Mooney faces were corrected to match that of the original NPT. Unless specified, the rest of this article refers to the modified computer-based test as the NPT with testing instructions similar to that described in the paper-based version (Mamiya *et al.*, 2016). For each NPT image, responses were classified as (i) Face—when a face was correctly identified in images embedded with a face; (ii) Noise—when a face was absent in the images; (iii) Pareidolia—when a face was identified in stimulus image without a face; and (iv) Missed—when a face was undetected in images that had a face embedded in them. Participants were asked to verbally respond to each image within 30 s and an experimenter recorded those responses separately by pushing a keystroke. All images were included into the statistical analysis with a separate set of three images used for training and calibration purposes.

### Eye-tracking and recording

An infrared eye-tracker system, Tobii Pro X3-120 (Tobii AB, Sweden), mounted on to a 24 inch, 1980 × 1080 pixels, 120 Hz monitor, captured eye movements and gaze data from the subjects. Participants were seated upright at a distance of 80 cm from the monitor display which had NPT images subtending a size of 700 × 700 pixels (width × height). A five-point calibration system was used with calibration error kept under 1.5°.

**Figure 1:**
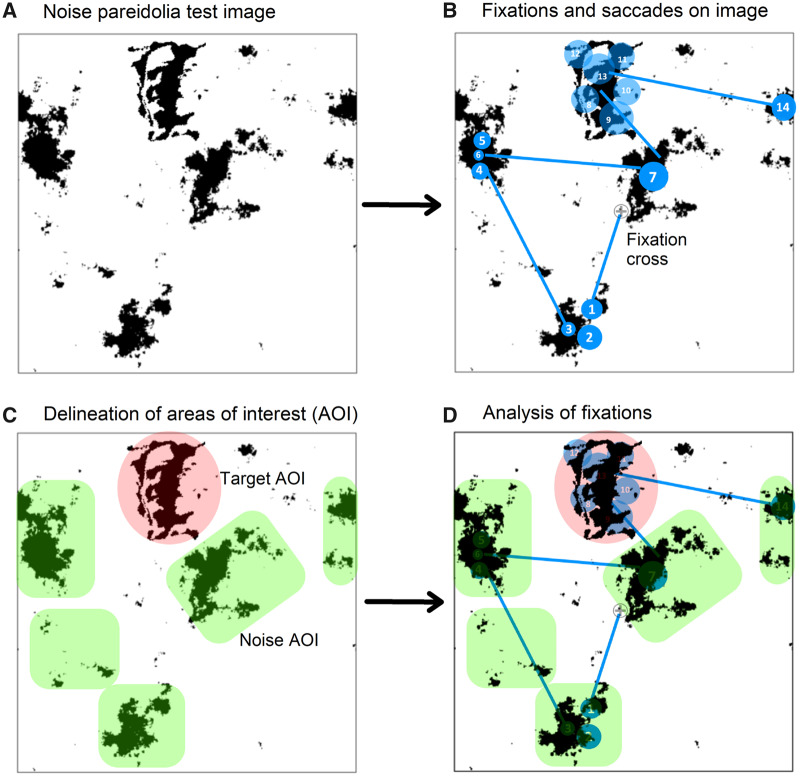
**Overview of methodology for eye-tracking in a NPT image.** (**A**) Example of a stimulus image with Mooney face embedded in the NPT. (**B**) Graphical representation of fixations (blue numbered bubbles) and saccades (blue solid lines) during visual scrutiny. The size of the bubble is proportional to the duration of fixation. Grey fixation cross shown is only for demonstration. (**C**) Prior demarcation of AOI on the stimulus presentation software—red ellipse for target faces and green polygons for noise. Most, if not all, target faces on the NPT were in the periphery removing the possibility of a central fixation bias (Tatler *et al.*, 2005). (**D**) Post-processing and analysis of fixation data were done with respect to mean values obtained from each AOI.

Each NPT image was preceded by a fixation cross for 2 s on the display screen on which subjects were asked to focus. Area of interest (AOI) for faces and for noise patches were demarcated beforehand on the presentation software using ellipses and polygons, respectively. This served to categorize fixations and saccades within the AOI’s for their subsequent analyses (Fig. 1). For each image, metrics from the gaze events and AOI data were subdivided into duration and count metrics. Duration metrics included (i) first fixation duration (FFD) (in milliseconds)—the duration of the first fixation on an AOI; (ii) total fixation duration (TFD) (in seconds)—the duration of all fixations within an AOI; and (iii) visit duration (VD) (in seconds)—the duration of all visits within an AOI. Count metrics comprised of (i) fixation count (FC)—number of times the subject fixates on an AOI and (ii) visit count (VC)—number of visit counts within an AOI. A visit, also known as dwell time, was defined as the period of time when a participant focuses on an AOI until they look away from that specific AOI. Visit duration consisted of at least one fixation but could include dozens, including the saccades, depending on the size of the AOI (Supplementary Fig. 2). These metrics were divided into early and late search for each stimulus image. For the entire experiment, healthy controls spent about 8.9 s (7.3, 9.4) [median (1st quartile, 3rd quartile)] per image whereas patients’ group spent 9.2 s (7.3, 12.4) per image. Early visual search was then set from stimulus onset till 5 s of the display and late search was binned from 5 s till the subjects’ response on the image. In general, Mooney face identification was not straightforward and participants took their time to identify faces, though a small number of stimuli that were tracked too fast or too slow were omitted from the analysis to maintain consistency.

### EEG recording and pre-processing

EEG data were recorded on a 32 channel cap-coordinate Biosemi system (ActiveTwo, BioSemi, Amsterdam, Netherlands) at 2 kHz sampling rate on ActiView software (LabVIEW, Neurospec AG, Switzerland). The recordings were then re-referenced to the average of all the electrode channels, pass filtered between 2 and 40 Hz and finally down-sampled to 500 Hz for independent component analysis. Independent component analysis was performed on the filtered data with a 400 ms shifting time window on continuous EEG recordings using the extended Infomax algorithm (Bell and Sejnowski, 1995; Makeig *et al.*, 1996). Independent components responsible for heartbeat, eye-blinks and muscle-related artefacts were isolated and rejected using visual inspection. Finally, the EEG was reconstructed by multiplying the non-artefactual components with the mixing matrix computed with the independent component analysis algorithm.

### Fixations for EEG analysis and epoching

To identify changes in the presaccadic interval, we classified eye-tracking data with respect to the saccade onset. This meant the ongoing fixation up until the start of the saccade was the time point for detection and evaluation of the fixation during the presaccadic interval. Constraints for inclusion of fixations as valid for EEG analysis were (i) fixation durations between 200 and 2000 ms, (ii) saccade durations <80 ms and (iii) valid fixations only up until the subjects’ verbal response to the image. Exclusion criteria were (i) fixations where blinks occurred within −300 to +300 ms relative to saccade onset and (ii) the first 700 ms after each image presentation to avoid stimulus onset-related potential. Saccades were divided into two groups with respect to saccade sizes—short saccades were those <4 degrees and those >4 degrees were classified as long saccades (Rayner, 2009). A matching procedure was performed to remove outlier fixations which did not match the saccade sizes using Mahalanobis distance (Nikolaev *et al.*, 2016). An arbitrary cut-off of 95th percentile for Mahalanobis distance was used to obtain fixations usable for EEG analysis (Supplementary Fig. 3).

With the above limits set, EEG data were then epoched from −200 ms to +50 ms with 0 being the start of the saccade onset. Artefacts from epochs exceeding 80 µV on either direction were rejected. Epochs were subsequently categorized with respect to subjects’ responses i.e. Face, Noise, Pareidolia and Missed. For each subject under each category, epochs were baseline corrected from −200 ms to −170 ms and further grand averaged to obtain cortical maps for evaluation of presaccadic potential. Scalp topographies for the full set of 32 channels were obtained to screen suitable electrodes for the study. Pertinent to our hypothesis, we focused our analysis on 10 important electrodes—five Frontal electrodes F3, Fz, F4, FC1 and FC2 and five Parietal electrodes P3, Pz, P4, PO3 and PO4. Our sensor locations were slightly anterior to the frontal eye fields in the superior frontal gyrus representing the frontal cortex, and in the posterior parietal cortex representing the parietal region. As with event-related potential studies, a minimum of at least 50 trial epochs were matched for statistical analysis (Luck, 2005). Within each response category, we identified the least number of trials and then a random number of trials were matched to keep the number same under all response conditions.

### Statistical analysis

Parkinson’s disease patients were dichotomized into Parkinson’s disease non-pareidolia type (PDnP) and Parkinson’s disease pareidolia type (PDP) using the original NPT which uses cut-off scores of 0–1 as non-pareidolics and ≥2 as pareidolics (Uchiyama *et al.*, 2015; Mamiya *et al.*, 2016). Due to the strong correlation towards the original paper-based NPT (Spearman’s coefficient, *N* = 33, adjusted *R*^2^ = 0.76), we adhered to the original NPT to classify our patients’ cohort. Reports for both eye-tracking and EEG analysis were generated based on correct (face and noise) and incorrect responses (pareidolias and missed). Eye-tracking studies typically have thousands of trials for fixations and saccade-related EEG analysis (Nikolaev *et al.*, 2016). Accordingly, the projected sample size needed for a three-group ANOVA with minimum detectable mean level = 0.3, expected standard deviation of residual = 0.15, alpha = 0.05 and power = 0.95 was *N* (participants) = 9.

For neuropsychological assessments, a Kruskal–Wallis test was done to reveal the differences between healthy controls (HC), PDnP and PDP groups. When significant, Dunn’s *post hoc* tests (for unequal groups) were performed. For comparisons within patient groups (PDnP and PDP), Student’s *t*-test or Mann–Whitney *U*-test was used.

For each eye-tracker metric, a mean value was calculated for each AOI. A two factorial repeated measures ANOVA was performed with between-subjects ‘Group’ (HC, PDnP and PDP) and within-subjects ‘Eye-tracker variable’ (i.e. either ‘Duration’ metrics comprising first fixation duration, TFD, VD or ‘Count’ metrics which included FC and VC) factors as independent variables. For significant ANOVA results, a *post hoc* multiple pairwise comparisons using Holm–Sidak adjustment was performed with an overall significance level set at 0.05. Since each response category of face, noise, pareidolia or missed were unbalanced and independent of each other, such of those categories where we observed significant interaction effects (*P* < 0.05), least-square means (LSM) and standard error of mean (SEM) of Group × Eye-tracker variable (highest *t*-value) were reported.

For EEG presaccadic activity, the mean amplitude of the time window between −100 ms and −20 ms from the start of the saccade was obtained. The 20 ms before the saccade onset was omitted to avoid the effects of a positive spike potential (Richards, 2003). We considered 2 main factors—‘Saccade size’ (long and short) and ‘Group’ (HC, PDnP and PDP)—for evaluation of fixation-related presaccadic potentials. In our formulation, we preselected a group of electrodes and to evaluate the topographical changes in amplitude differences, we first used the frontal and parietal groups of electrodes as dependent variables for a multivariate ANOVA model (MANOVA) (Murray *et al.*, 2008; Nikolaev *et al.*, 2013). To test assumption for use of MANOVA model, we performed correlational analysis on frontal and parietal presaccadic amplitudes, limits set between *r* = 0.3–0.6 (moderate correlation). Outliers were removed using Mahalanobis distance, and visualized scatterplots for outliers on each parameter (Soto *et al.*, 2009). Only when MANOVA results were significant, a univariate ANOVA was performed between the ‘Saccade sizes’ and ‘Group’. Normality of the data was tested using Lilliefors corrected Kolmogorov–Smirnov test with the normality statistic set to 0.05. For responses that did not achieve normality, non-parametric ANOVA was performed (Ghasemi and Zahediasl, 2012). Statistical significance for ANOVA was set to *P* < 0.05 with subsequent *post hoc* test performed for each significant dependent variable. Full details of statistical testing are shown in Supplementary data. Offline analysis, statistics and graphing of data were done using Matlab 2017b (The Mathworks Inc, Natick, MA). EEG pre-processing was implemented with an open-source toolbox, Brainstorm, on Matlab 2017b, available at http://neuroimage.usc.edu/brainstorm (Tadel *et al.*, 2011).

### Data availability

Data analysed in this study will be made available from the corresponding author upon reasonable request.

## Results

### Demographic and neuropsychological assessment

Demographic and neuropsychological details for HC, PDnP and PDP groups are shown in Table 1. We observed patients with pareidolias (PDP) were older than those without pareidolias (PDnP). Except for age, there were no other significant differences in demographic characteristics among the two patient groups.

With respect to neuropsychological assessment scores, frontal assessment battery, Montreal cognitive assessment and Benton’s judgement line orientation test were sensitive to differentiate between HC and patients but not within the patients’ groups (*post hoc* tests between PDnP and PDP). No significant differences were observed with MMSE and Japanese adult reading test scores.

For the NPT, two patients performed only a part of the test (65 and 40 images, respectively) and were unable to continue due to back pain. Data for these two patients were treated as is without imputing missing values. PDP group showed considerably higher pareidolic counts in the modified NPT when compared to HC and PDnP groups. However, for missed face responses, no such differences were observed among HC and patient groups.

### Eye-tracking results—early and late search dynamics

**Figure 2:**
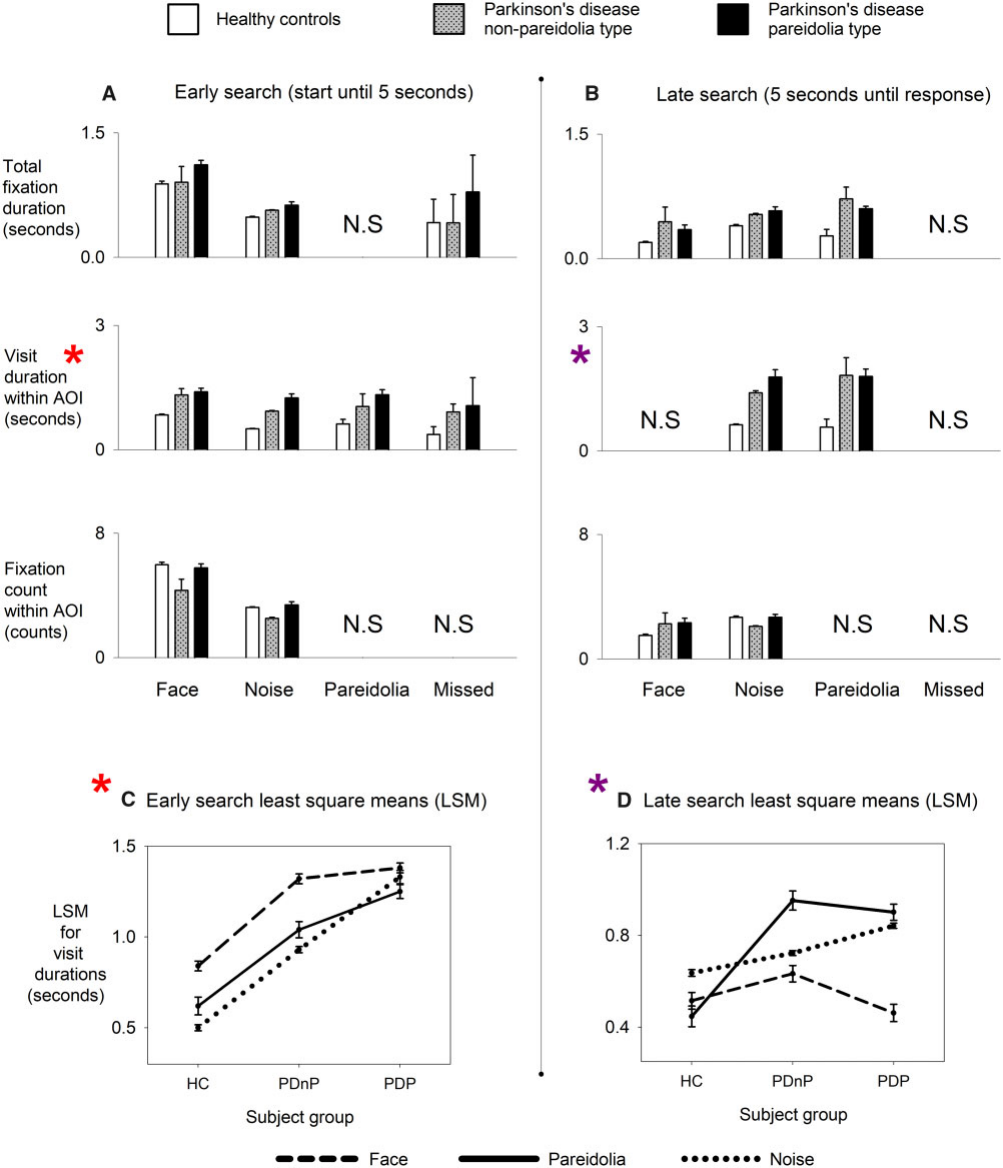
**Eye movement metrics for early and late search.** N.S = not significant (*post hoc* test), LSM = least-square means, HC = healthy controls (*N* = 12), PDnP = Parkinson’s disease non-pareidolia type (*N* = 11), PDP = Parkinson’s disease pareidolia type (*N* = 10). Grouped bar plot for (**A**) Early and (**B**) Late visual search metrics shows mean and SD of AOI’s for—total fixation duration, visit duration and fixation count on *Y*-axis; and categories—face, noise, pareidolia and missed responses on *X*-axis. Error plots of least-square means from visit durations (asterisks) are shown in **C** for early search and **D** for late search. During early search, visit durations were proportional to the saliency exhibited by the images indicating steady increase of cognitive and visuo-spatial demands for the stimuli, seen as HC > PDnP > PDP. During late search, pareidolia responses showed similar visit durations in patients’ groups due to the temporal independence of fixation characteristics on attentional requirements. Missed responses were omitted since raw NPT scores were not significant among the control and patient groups.

Full eye-movement characteristics are summarized in Fig. 2. Results for early search are shown in Table 2 and in Fig. 2A. Both duration and count metrics during early search showed significant interaction effects (*P*-values < 0.001) for each response category. Inspecting LSM for each response, PDP group showed longer fixation and visit durations on AOI’s compared to PDnP group, which in turn were longer than HC group. While Parkinson’s disease patients spent nearly the same duration on target AOI’s to identify faces, PDP group dwelled significantly longer than PDnP group for pareidolic responses (by about 200 ms). Fixation counts in general were higher for faces than for noise suggesting higher convergence of focus towards salient stimuli, though were inconsequential between the groups when considering their mean values.

**Table 2:** Summary of eye-tracker metrics during early visual search (0–5 s)

Category		Main effects		Interactions	Least-square means (of significant interactions)					
		Subjects	Variables		Total fixation duration			Visit durations		
		Group (HC, PDnP, PDP)	Durations (FFD, TFD, VD)	Group × Durations	HC	PDnP	PDP	HC	PDnP	PDP
**Duration metrics**	Face	F_(2,58)_ = 31.37, *P < 0.001*	F_(2,58)_ = 1226.32, *P < 0.001*	F_(4,58)_ = 36.04, *P < 0.001*	0.88 ± 0.03	0.91 ± 0.03	1.10 ± 0.03	0.84 ± 0.03	1.32 ± 0.03	1.38 ± 0.03
	Noise	F_(2,56)_ = 169.34, *P < 0.001*	F_(2,56)_ = 1202.57, *P < 0.001*	F_(4,56)_ = 109.48, *P < 0.001*	0.48 ± 0.02	0.57 ± 0.02	0.72 ± 0.04	0.50 ± 0.02	0.93 ± 0.02	1.33 ± 0.04
	Pareidolia	F_(2,40)_ = 20.74, *P < 0.001*	F_(2,40)_ = 205.80, *P < 0.001*	F_(4,40)_ = 14.34, *P < 0.001*	N.S	N.S	N.S	0.62 ± 0.05	1.04 ± 0.05	1.25 ± 0.04
	Missed	F_(2,56)_ = 3.62, *P = 0.039*	F_(2,56)_ = 40.75, *P < 0.001*	F_(4,56)_ = 6.46,*P < 0.001*	0.42 ± 0.08	0.41 ± 0.08	1.01 ± 0.16	0.37 ± 0.08	0.86 ± 0.09	1.29 ± 0.16
		**Group** (**HC**, **PDnP**, **PDP**)	**Counts** (**FC**, **VC**)	**Group** **×** **Counts**	**Fixation Counts**					
					**HC**	**PDnP**	**PDP**			
**Count metrics**	Face	F_(2,29)_ = 50.24, *P < 0.001*	F_(1,29)_ = 4265.24, *P < 0.001*	F_(2,29)_ = 35.99, *P < 0.001*	5.97 ± 0.1	4.33 ± 0.1	5.81 ± 0.11			
	Noise	F_(2,29)_ = 207.46, *P < 0.001*	F_(1,29)_ = 8991.28, *P < 0.001*	F_(2,29)_ = 119.73, *P < 0.001*	3.23 ± 0.03	2.52 ± 0.03	3.47 ± 0.03			
	Pareidolia	F_(2,20)_ = 2.22, *P* = 0.134	F_(1,20)_ = 734.83, *P < 0.001*	F_(2,20)_ = 5.26, *P = 0.015*	N.S	N.S	N.S			
	Missed	F_(2,29)_ = 1.99, *P* = 0.154	F_(1,29)_ = 76.76, *P < 0.001*	F_(2,29)_ = 3.63, *P = 0.039*	N.S	N.S	N.S			

Least-square means are reported with standard errors (in seconds).

FC = fixation count; FFD = first fixation duration; N.S = (*post hoc* test) not significant; TFD = total fixation duration; VC = visit count; VD = visit duration.

Significant *P*-values are italicized.

Table 3 and Fig. 2B show the statistical results for late visual search. Notably, patient groups showed longer fixation and visit durations when compared to HC. Visit durations for pareidolia response, though significant, were remarkably similar for PDnP and PDP groups (difference of <50 ms). Fixation counts were lower during late search most likely due to response occurrence at end of the stimulus. We found critical changes for missed responses during early and late search but avoided their interpretations since raw NPT scores did not show any significant differences [H(2) = 3.05, *P* = 0.21] between the three groups.

**Table 3:** Summary of eye-tracker metrics during late visual search (5 s until response)

Category		Main effects		Interactions	Least-square means (of significant interactions)					
		Subjects	Variables		Total fixation duration			Visit durations		
		Group (HC, PDnP, PDP)	Durations (TFD, VD)	Group × Durations	HC	PDnP	PDP	HC	PDnP	PDP
**Duration metrics**	Face	F_(2,29)_ = 6.69, *P = 0.004*	F_(1,29)_ = 1669.17, *P < 0.001*	F_(2,29)_ = 113.76, *P < 0.001*	0.19 ± 0.04	0.44 ± 0.04	0.33 ± 0.04	N.S	N.S	N.S
	Noise	F_(2,24)_ = 24.36, *P < 0.001*	F_(1,24)_ = 9505.08, *P < 0.001*	F_(2,24)_ = 60.17, *P < 0.001*	0.40 ± 0.01	0.53 ± 0.01	0.60 ± 0.01	0.62 ± 0.01	0.72 ± 0.01	0.84 ± 0.01
	Pareidolia	F_(2,20)_ = 32.71, *P < 0.001*	F_(1,20)_ = 349.85, *P < 0.001*	F_(2,20)_ = 3.87, *P = 0.038*	0.27 ± 0.04	0.72 ± 0.04	0.65 ± 0.03	0.45 ± 0.04	0.95 ± 0.04	0.90 ± 0.03
	Missed	F_(2,29)_ = 2.67, *P* = 0.086	F_(1,29)_ = 27.67, *P < 0.001*	F_(2,29)_ = 2.97, *P* = 0.067	N.S	N.S	N.S	N.S	N.S	N.S
		**Group** (**HC**, **PDnP**, **PDP**)	**Counts** (**FC**, **VC**)	**Group** **×** **Counts**	**Fixation counts**					
					**HC**	**PDnP**	**PDP**			
**Count metrics**	Face	F_(2,29)_ = 4.62, *P = 0.018*	F_(1,29)_ = 378.41, *P < 0.001*	F_(2,29)_ = 14.70, *P < 0.001*	1.50 ± 0.1	2.25 ± 0.1	2.37 ± 0.1			
	Noise	F_(2,24)_ = 84.88, *P < 0.001*	F_(1,24)_ = 5722.18, *P < 0.001*	F_(2,24)_ = 63.65, *P < 0.001*	2.70 ± 0.03	2.09 ± 0.03	2.74 ± 0.03			
	Pareidolia	F_(2,20)_ = 2.93, *P* = 0.076	F_(1,20)_ = 682.20, *P < 0.001*	F_(2,20)_ = 20.81, *P < 0.001*	2.00 ± 0.11	2.76 ± 0.1	2.75 ± 0.09			
	Missed	F_(2,29)_ = 1.73, *P* = 0.195	F_(1,29)_ = 36.56, *P < 0.001*	F_(2,29)_ = 7.92, *P = 0.002*	0.89 ± 0.26	0.72 ± 0.26	2.76 ± 0.27			

Least-square means are reported with standard errors (in seconds).

FC = fixation count; FFD = first fixation duration; N.S = (*post hoc* test) not significant; TFD = total fixation duration; VC = visit count; VD = visit duration.

Significant *P*-values are italicized.

To sum up early and late search, changes in visit durations were significant during early visual search for PDP group with the effects plateauing during the late phase for both patient subgroups. A summary comparison of adjusted means of significant interactions effects for visit durations is outlined in Fig. 2C for early search and Fig. 2D for late search.

### EEG results—presaccadic amplitudes for long and short saccades

**Figure 3:**
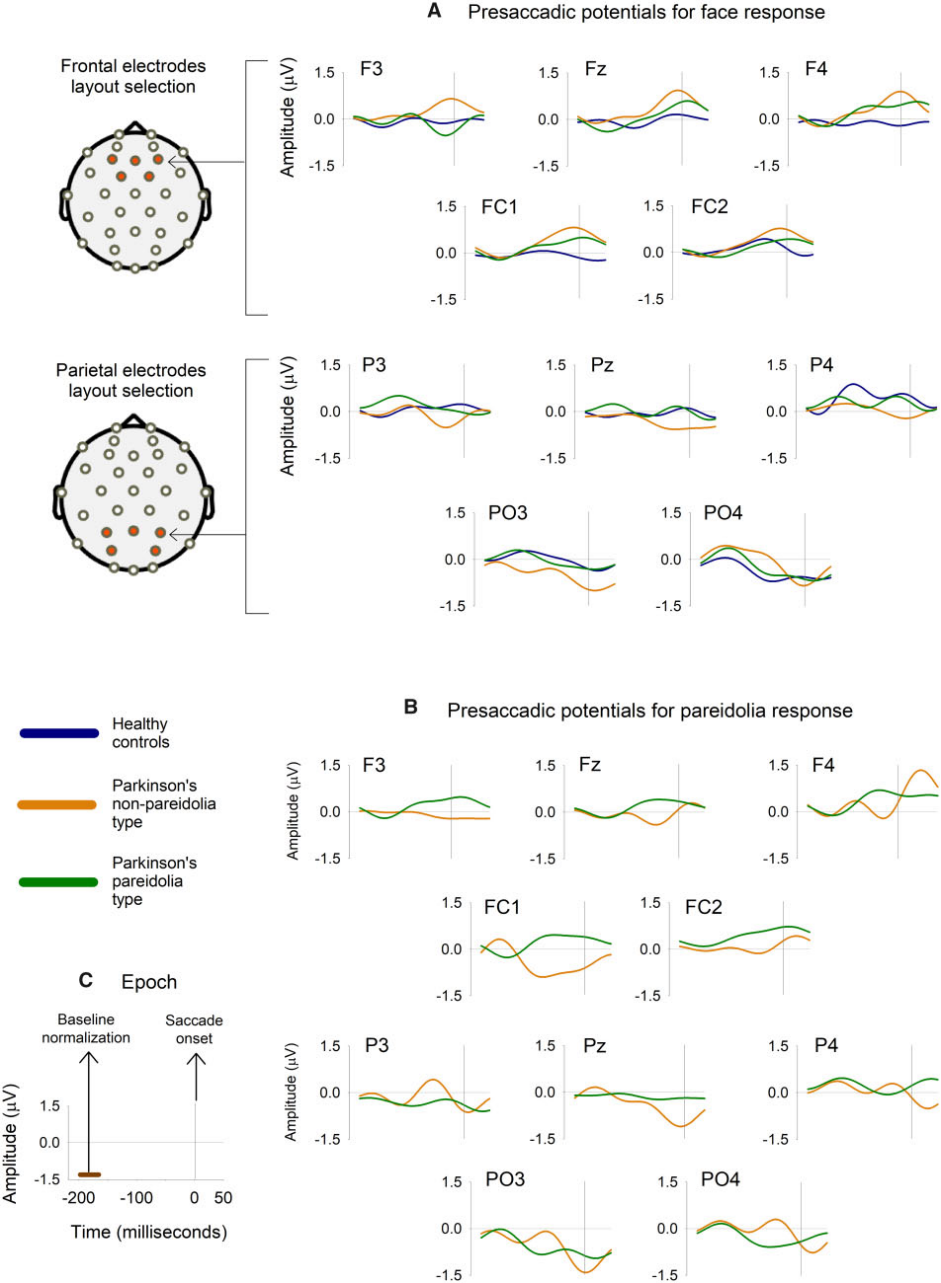
**Grand averaged EEG waveforms for faces and pareidolia response.** This figure shows presaccadic potentials for (**A**) face response and (**B**) pareidolia response. The averaged fixation potentials were obtained from re-referenced EEG for five frontal and five parietal electrodes as shown. Presaccadic activity is a slow potential, peaking at around 100 ms before saccade onset positive for targets and negative for distractors. (**C**) Bottom left, shows epoch length used for analysis. Epochs from each electrode were baseline normalized between −200 and −170 ms and presaccadic amplitude was calculated as mean values between −100 and −20 ms with respect to saccade onset at time 0.

There were two dropouts within the HC group (due to technical complications) bringing the total number of tested subjects to HC = 10, PDnP = 11 and PDP = 10 for EEG analysis. For evaluation of presaccadic potentials, we obtained a grand total of HC = 4053, PDnP = 5297 and PDP = 6425 valid fixations after performing the matching procedures (Supplementary Fig. 3). PDP group had significantly higher total saccade count (166 447) when compared to HC (99 696) or PDnP group (101 705). HC group had too few valid fixations for pareidolia = 0 (0, 12) and for missed responses = 9 (1, 14), to be included in the analysis. As a result, for face and noise category, all three groups were compared whereas pariedolia and missed responses were compared only among Parkinson’s disease patients. Figure 3 shows grand averaged means of presaccadic potentials for face (Fig. 3A) and for pareidolia (Fig. 3B) response.

**Figure 4:**
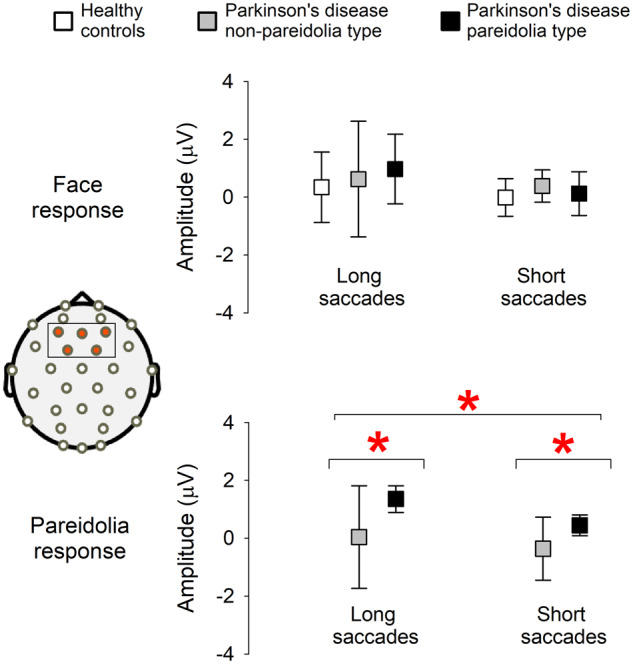
**Comparison of presaccadic amplitudes in frontal electrodes.** For face response, HC (*N* = 10), PDnP (*N* = 11) and PDP (*N* = 10). For pareidolic responses PDnP (*N* = 9) and PDP (*N* = 10). Each error plot shows the grouped scatter mean with SD between long and short saccades. Red asterisks show significant (*P* < 0.05) univariate ANOVA differences for saccade sizes and between patient groups PDnP and PDP. Patients susceptible to pareidolias showed a significantly higher frontal presaccadic activity irrespective of saccade sizes.

Table 4 details the summary results for EEG analysis. With the exception of missed responses, two-way ANOVA showed significant effect of saccade sizes for face, noise and pareidolia responses in the parietal electrodes. After allowing effects of saccade sizes, presaccadic potentials for face response were similar for all three subject groups. Noise responses were statistically significant in parietal electrodes with presaccadic amplitudes being more negative in HC group when compared to either PDnP or PDP groups (LSM, HC = −0.93 µV ± 0.15, PDnP = −0.41 µV ± 0.14 and PDP = −0.38 µV ± 0.15). Of significance are results for pareidolia responses in frontal electrodes where *post hoc* tests showed positive frontal presaccadic potentials in PDP group when compared to PDnP group (PDnP = −0.27 µV ± 0.25 and PDP = 0.9 µV ± 0.22). Figure 4 shows a comparison of presaccadic amplitudes for face and pareidolia responses. Results for all response variables are shown in Supplementary Fig. 4 and Tables 1 and 2.

**Table 4:** Summary of EEG results—presaccadic amplitudes comparison between frontal and parietal electrodes

Category	MANOVA	Two-way ANOVA			
	Frontal electrodes versus Parietal electrodes	Main effects			
		Frontal electrodes		Parietal electrodes	
		Saccade size (long, short)	Group (HC, PDnP, PDP)	Saccade size (long, short)	Group (HC, PDnP, PDP)
Face	F_(2,58)_ = 1.82, P = 0.170, Wilk’s λ = 0.9	N.S	N.S	N.S	N.S
Noise	F_(2,59)_ = 3.93, *P = 0.025*, Wilk’s λ = 0.8	F_(1,56)_ = 7.06, *P* ***=****0.010*	N.S	F_(1,56)_ = 7.28, *P* ***=****0.009*	F_(2,56)_ = 4.34, *P* ***=****0.018*
Pareidolia	F_(2,33)_ = 3.82, *P = 0.032*, Wilk’s λ = 0.8	F_(1,32)_ = 14.39, *P* *<* *0.001_n.p_*	F_(1,32)_ = 23.16, *P* *<* *0.001_n.p_*	F_(1,32)_ = 12.31, *P* ***=****0.001_n.p_*	N.S
Missed	F_(2,39)_ = 0.46, P = 0.635, Wilk’s λ = 0.9	N.S	N.S	N.S	N.S

n.p. = non-parametric test; N.S. = not significant.

Significant *P*-values are italicized.

## Discussion

Our report is the first study to clarify the course of pareidolic illusions in Parkinson’s disease patients through the NPT. Using synchronized eye-tracking and EEG, we found that (i) the NPT is able to capture visuo-perceptual inadequacies in patients despite variations in cognitive assessment, (ii) Parkinson’s disease patients dwell longer on images to converge attention to pareidolic stimuli that is proportional to their visuo-perceptual deficit, which affects early and late visual scrutiny, and (iii) patients prone to pareidolias show increased frontal activation irrespective of their saccade sizes, which reflects the allocation of attention to irrelevant targets, resulting in pareidolic phenomena. In the following sections, we demonstrate these features of top-down visual processing to explain the effects and mechanism of pareidolias in patients susceptible to such visual illusions.

### Pareidolias and visuo-perception

As a test to measure visuo-perception in our patients, we confirmed that the NPT can clearly distinguish between patients with and without pareidolic tendencies. The NPT requires a subject to search, select and interpret ambiguous stimuli, which requires a knowledge-based integration of face processing (Schwartzman *et al.*, 2008). To perform such tasks, in early and moderate Parkinson’s disease patients, the integrity of the frontal cortex (dorsolateral, frontostriatal and frontal eye fields) is presumed to be important, as any deficits are thought to be associated with the loss of working memory, visual perception and higher-order executive function (Bradley *et al.*, 1989; Owen, 2004). Specifically, visuo-perceptual discrepancies are an eventuality of global cortical network dysfunction, and the frontal cortex is frequently suggested to be dysfunctional by the time hallucinations appear (de la Fuente-Fernández, 2011; Shine *et al.*, 2011). As pareidolias precede hallucinations, we speculate that patients who experience pareidolias create an abnormal target-driven competition for attention from weak stimuli owing to an abnormality of the dorsal fronto-parietal network (Shine *et al.*, 2012, 2014). Despite the cognitive similarities among pareidolic and non-pareidolic patients in our group, differences in their visuo-perception represent a behavioural contrast caused by the altered integration of non-salient stimuli to expectation, preparation, and the selection of targets (Connor *et al.*, 2004; Shine *et al.*, 2014).

### Pareidolias and eye movement characteristics

The second arm of this behavioural representation of the NPT consisted of tracking eye movements. Phenomenologically, eye movement characteristics during the early phase of visual scrutiny represent global search mechanisms, whereas those during the late phase refer to local convergence to the target. This forms the premise of free viewing, wherein visual scrutiny dynamically converges from a bottom-up to a top-down process reflected by eye movements that perform a sweeping search of the image to finally pin-point the target (Tatler *et al.*, 2005; Fischer *et al.*, 2013).

During the early search, we found that when salient features within the image decreased (such as in noise images), the visit durations were also relatively lower, with pareidolias resting right between face (highly salient) and noise (less salient) stimuli. This order of increasing fixation lengths (shown as visit durations) highlights how visual processing systems steadily augment information in the NPT. By definition, visit duration comprises both fixations and saccades within a particular area of interest, and the process of visual signal integration is closely connected to the saccadic activity that these fixations depend upon (Tatler *et al.*, 2006). In susceptible patients, pareidolias necessitated an increase in saccade generation to ideally integrate visual information from noisy areas that appear relevant. This pattern of visit durations confirms findings from another study showing that Parkinson’s disease patients tend to generate extra saccades for complex images, which depends on the saliency of the image (Matsumoto *et al.*, 2011). This may well be an aspect signifying early attention and fixation behaviour (Cajar *et al.*, 2016), but could also be an epiphenomenon caused by aberrant saccade generation, with or without decreased smooth pursuit or hypometric saccades in Parkinson’s disease (Matsumoto *et al.*, 2011; Archibald *et al.*, 2013; Terao *et al.*, 2017).

Whereas stimulus-driven fixations are used for early search responses, late search responses are top-down consequences of free viewing (Fischer *et al.*, 2013; Moore and Zirnsak, 2015). Patients with pareidolias who dwell longer on an area of interest demonstrate a delay in the pooling of attentional resources to focus on a target (Matsumoto *et al.*, 2011; Archibald *et al.*, 2013). Parkinson’s disease patients conflate noisy perceptions as targets, and our cohort showed no substantial differences between the non-pareidolic and pareidolic groups during the late search. This is in line with a previous report (Matsumoto *et al.*, 2011) suggesting the independence of fixation metrics with respect to focus of fixation in Parkinson’s disease patients.

Taken together, regarding eye-tracking behaviour during visual scrutiny, patients with pareidolias were unable to maintain the cognitive demands of the NPT, which forces additional shifts of attention creating an overdependence of dorsal attention networks to decipher pareidolic stimuli, irrespective of the temporal constraints (Shine *et al.*, 2011). The top-down modulation of these networks therefore appears to play an important role given its contributions in fixation lengths and saccades towards attentional requirements in perceptual decision-making tasks (Hwang *et al.*, 2009).

### Pareidolias and presaccadic potentials

The next point is clarifying the role of oculomotor planning and the transfer of attention to salient stimuli via presaccadic potentials. Patients with pareidolias showed a marked increase in frontal presaccadic potentials irrespective of saccade size, suggesting that both exploration and localization were affected when pareidolias were observed. During exploration, the prefrontal cortex and frontal eye fields contribute to presaccadic activity for purposeful goal-directed saccades (Funahashi, 2014). A positive presaccadic potential guided towards a target represents the planning of motor behaviour (Richards, 2003), and consequently a shift in visual attention (Heeman *et al.*, 2014), indicating top-down facilitation of anticipatory stimuli in the frontal cortex (Bressler *et al.*, 2008). Presaccadic amplitudes of long saccades involved in the ‘selection’ of ambiguous stimuli partly explain the pareidolic misperceptions evoked by frontal eye fields during the covert shift of attentional systems to encode visual information (Gutteling *et al.*, 2010). In addition, our findings also point to a ‘reinforcement’ of this selection in the frontal areas, which appears to precipitate the effect of pareidolias. In the brain, during localization of a target, the accumulation of visual information by short saccades remaps visual stimuli to salient stimuli (Smith and Schenk, 2012). The change in presaccadic amplitudes seen in our study between the pareidolic and non-pareidolic groups illustrate the strength of successful encoding that occurs with respect to short saccades in these groups. Both long and short saccades are necessary to identify a relevant target (Unema *et al.*, 2005), and these differences therefore signify alterations in the planning, transfer of attention and visual encoding of patients with pareidolias.

Regardless of these findings in the frontal cortex, we speculate that the phenomenon of pareidolic misperception may involve wider areas of the brain, including the parietal cortex. When imprinting salient visual information, the lateral intraparietal area in the posterior parietal cortex acts as a priority map that ‘selects’ relevant stimuli to encode oculomotor activity (Bisley and Goldberg, 2010). The posterior parietal cortex, which is a part of the dorsal attentional network, along with occipital visual areas, uses short-term memory-guided sequences to transfer attention to a target (Parks and Corballis, 2008). For pareidolic responses, presaccadic activity in the parietal cortex was seen to depend on saccade sizes, making it slightly complex to unequivocally define its effect on our patients. Despite the role of lateral intraparietal area in the planning and coordination towards covert attention (Bisley and Goldberg, 2010), our findings of the frontal electrodes do not obviate the top-down integration of information by the parietal cortex that may be taking place simultaneously in the presaccadic phase (Mathôt and Theeuwes, 2011).

Pareidolias share the same principle as complex hallucinations, and this over-activation of presaccadic potentials in the frontal cortex is the key characteristic of patients who are likely to hallucinate. Our task-based approach to estimate fixation-related potentials establishes the effects of abnormal visual processing in such patients, adding valuable interpretations to results previously reported in a series of studies that have utilized the NPT (Yokoi *et al.*, 2014; Uchiyama *et al.*, 2015; Nishio *et al.*, 2017).

### Limitations

There are some limitations to this study. Although we did not use faces from the original NPT, we expected that our modifications of Mooney face stimuli would increase the specificity of the test among Parkinson’s disease patients, as they miss faces frequently owing to impaired unfamiliar face recognition (Dewick *et al.*, 1991; Kida *et al.*, 2007). The dorsal network, which is responsible for the above function, is usually affected in early and moderate Parkinson’s disease (Flowers and Robertson, 1995). However, the healthy controls missed faces just as frequently as the patients. We believe there were two reasons to this. First, our attempt to avoid the floor-effect in the patients may have resulted in some images being too difficult. Second, owing to the length of the experiment, some target faces were almost always missed in the latter part of the experiment.

One patient who had a positive history for visual hallucinations (presence type, for the past 1 year) also demonstrated pareidolic behaviour (a score of 52 pareidolias in the NPT). Although pareidolias in patients form a part of the minor hallucinatory process, we believe the effect was exaggerated in this patient. Owing to their non-specific nature, we also did not report specific crucial subjective experiences of other patients describing noisy patches on NPT as dogs or birds in the images. We believe these features are still relevant to the effects of illusions, and more data are necessary to understand the individual effects.

The saliency of Mooney face stimuli was not trivial, and considering the length of the experiment, a certain degree of temporal variability of the EEG was unavoidable (Marathe *et al.*, 2014). There are numerous saccade problems in Parkinson’s disease and their effects are invariably present in all eye-tracking studies (Hood *et al.*, 2007; Matsumoto *et al.*, 2011; Terao *et al.*, 2017). Our interest was to observe the changes in eye-fixation-associated EEG potentials, and we were careful to interpret saccade behaviour only with respect to visit durations, which incorporated fixations along with saccades.

Furthermore, there may be gender differences (Crucian and Okun, 2003; Proverbio and Galli, 2016) although such differences in a visual search experiment are expected to be minor. We did not systematically study the effects of age and levodopa dose, which may contribute to an effect on fixation characteristics (Hood *et al.*, 2007). Addressing these concerns would be beneficial in the future, using fewer test images and assessing how specific treatments would affect the performance on the NPT using longitudinal follow-up studies.

## Conclusions

Pareidolias on their own are not pathologic (Liu *et al.*, 2014). However, in a subset of Parkinson’s disease patients, pareidolias may contribute to the development of hallucinatory features, as shown by the identification of eye movement behaviours and cortical brain activity that accompany visuo-perceptual deficits. The culmination of such deficits is that patients experiencing pareidolias are unable to disregard less meaningful stimuli owing to abnormal top-down modulation of visual processing.

## Supplementary Material

fcaa073_Supplementary_Data

## Abbreviations

AOI =: area of interest
HC =: healthy controls
LSM =: least square means
NPT =: noise pareidolia test
PDnP =: Parkinson’s disease non-pareidolia type
PDP =: Parkinson’s disease pareidolic type
