# Supplementary material for: Ocular fixations and presaccadic potentials to explain pareidolias in Parkinson’s disease
Source: Brain Commun. 2020 Jun 4;2(1):fcaa073. doi: 10.1093/braincomms/fcaa073 (PMC7425388; doi:10.1093/braincomms/fcaa073)
Supplement: fcaa073_Supplementary_Data [file fcaa073_supplementary_data.pdf]

Supplementary Data

Supplementary Methodology

Eye tracker settings and co-registration of EEG with eye-tracker

An infrared eye-tracker system, Tobii Pro X3-120 (Tobii AB, Sweden), was used to obtain eye movements and gaze data from the participants. The eye tracker was known to have good accuracy for binocular, free-viewing tasks while allowing some tolerable head movements making it particularly feasible among our patient group wherein the use of a chin-rest was not conducive. A default velocity-threshold identification classification algorithm was used to define fixation and saccades based on the velocity of the directional shifts of the eye (Olsen and Matos, 2012). Fixations were defined as a cluster of raw eye-tracker data points with a velocity threshold set at 30 degrees/second, minimum fixation duration of 60ms and interpolation of data points occurring within 70ms (Komogortsev *et al.*, 2010).

Synchronization between eye-tracker and EEG was achieved using a photodiode placed on the edge of the stimulus presentation monitor which sent trigger information (stimulus onset) through a TTL (transistor-transistor logic) pulse to the EEG receiver connected to the EEG recording laptop. Time stamps obtained from the eye-tracker were then adjusted for any lag and then integrated in to the EEG file during preprocessing to obtain co-registered data (Xue *et al.*, 2017). Temporal accuracy of eye-tracker and EEG run times was verified to have negligible lag, calculated using Pearson's correlation coefficient.

Supplementary Figures

Supplementary Figure 1 – Original versus modified noise pareidolia test

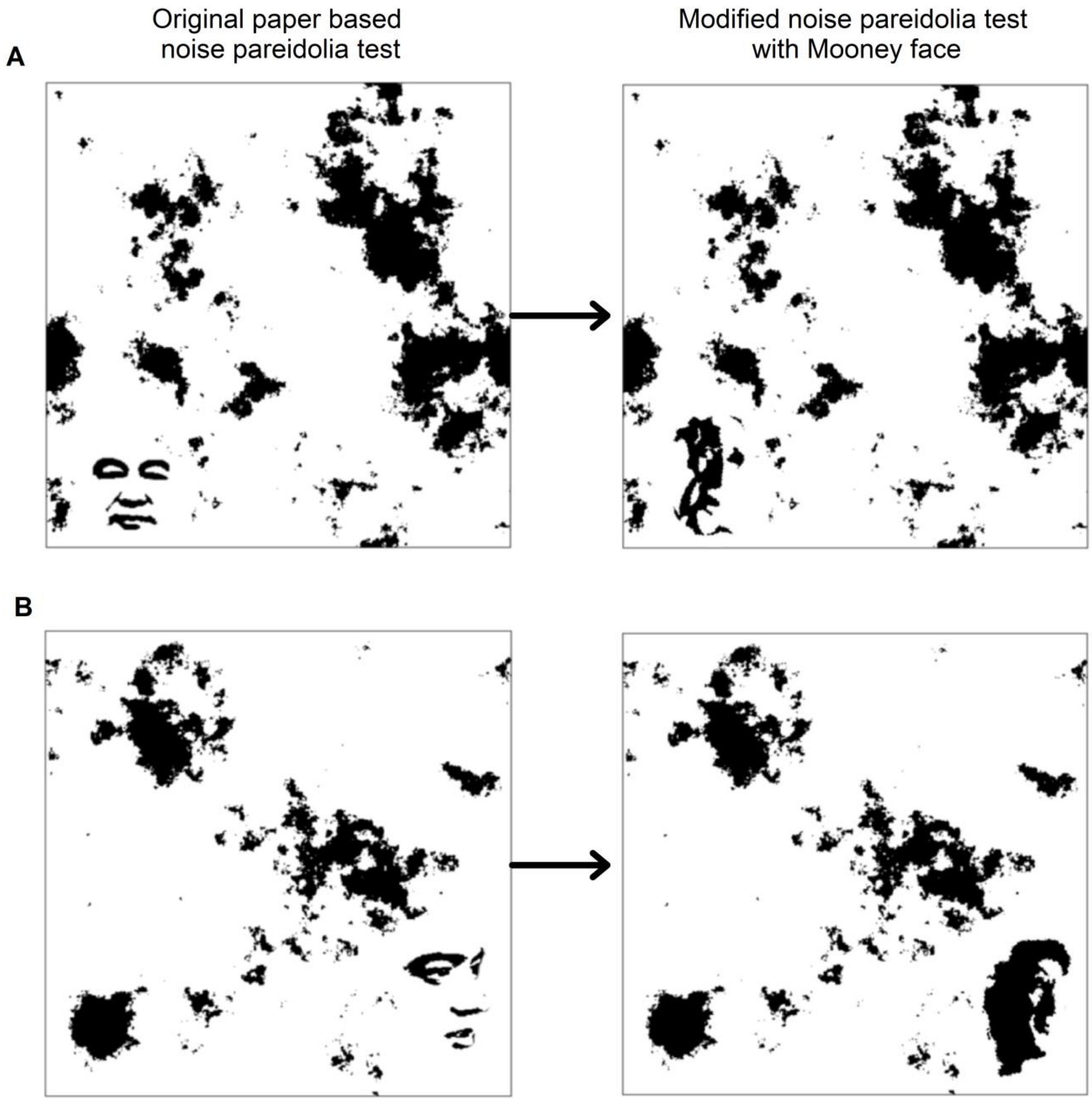

Supplementary Figure 1 shows two examples in panel A and panel B of faces from original noise pareidolia test replaced by more ambiguous Mooney faces (shown on the right). The replaced Mooney faces had the same dimensions and features (direction, angle, shape, emotional neutrality) as that of the original test.

Supplementary Figure 2 – Eye metrics calculations

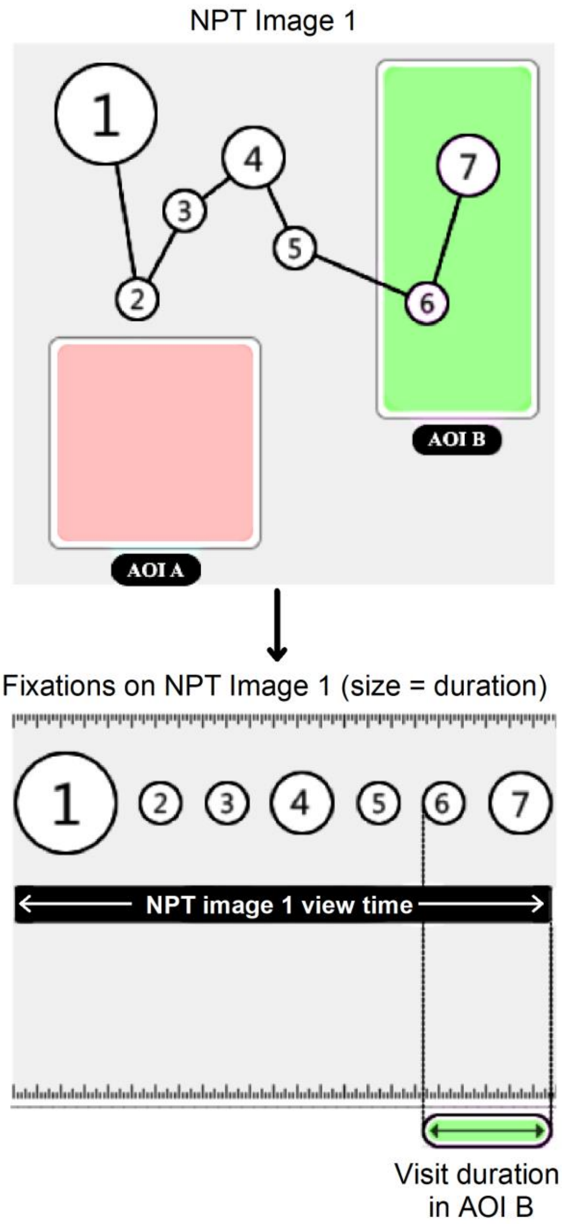

Supplementary Figure 2 shows a graphical representation outlining the calculation of eye metrics (redrawn from Tobii Pro Studio presentation software). For an example noise pareidolia test (NPT) image 1 (top), areas of interest (AOI) are shown as AOI A and AOI B. Numbers 1 to 7 represent fixations on the NPT Image 1. Size of the bubble is the fixation duration. First fixation duration is the size of fixation number 1. For AOI B, Fixation count = 2 (numbers 6 and 7), Total fixation duration = size of bubble 6 + size of bubble 7, and visit duration = size of bubble 6 + size of bubble 7 + the saccade connecting bubble 6 and bubble 7.

Supplementary Figure 3 – Methodology for obtaining fixations for EEG analysis

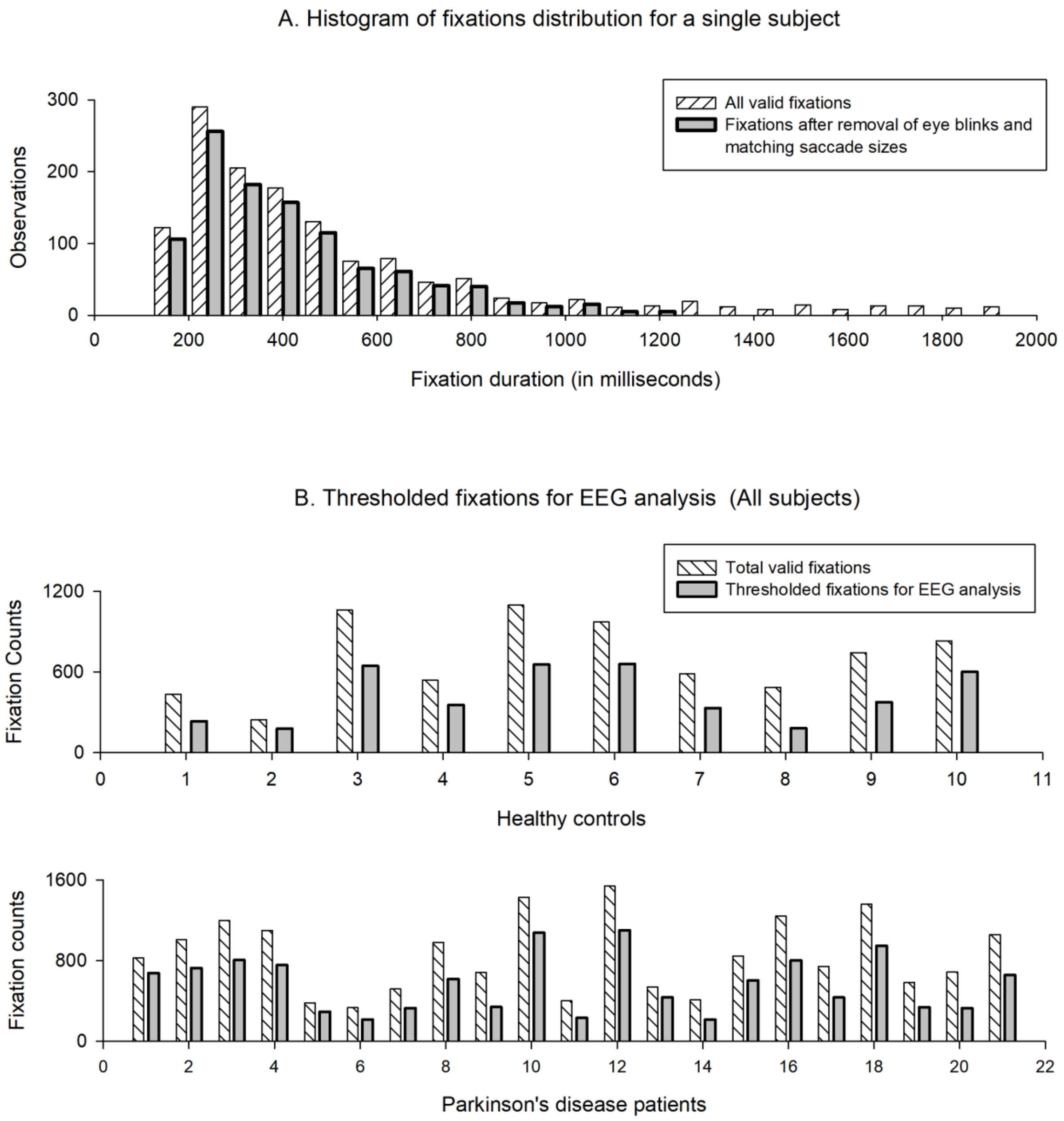

Supplementary Figure 3A - All fixations from the eye tracker (striped-bars) shown are those 'after' satisfying the inclusion criteria of fixation duration between 200ms to 2000ms. After removing segments of EEG of eye-blinks, saccades were matched with their fixation durations using saccade sizes by calculating the Mahalanobis distance. A 95th percentile cutoff was arbitrarily used to remove all fixations that did not match. Eye movement distortions are always

present in the EEG. Matching eye movement with respect to saccade sizes balances the distributions of eye movement features making epoch analysis meaningful (Nikolaev *et al.*, 2016). Saccade sizes defined from the Tobii Pro eye tracking system takes into account the direction of the eye movement and hence was sufficient to match with the fixation duration. Solid grey bars are fixations that were used for EEG analysis.

Supplementary Figure 3B - Shows the above described procedure to obtain fixations for EEG analysis from all participants, healthy controls (N = 10) and Parkinson's disease patients (N = 21).

Supplementary Figure 4 –Presaccadic amplitudes for all response categories

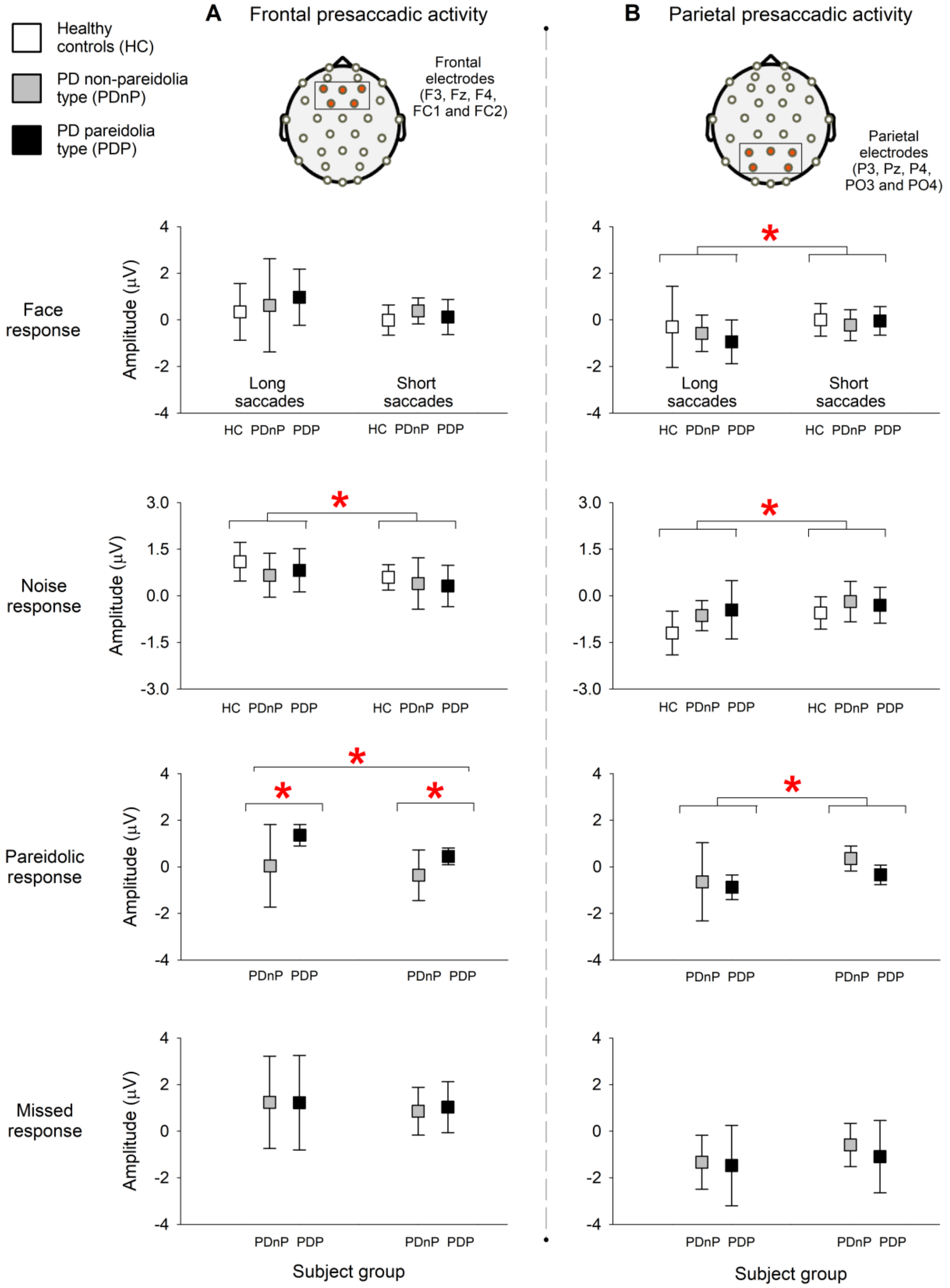

Supplementary Figure 4. Each error plot shows the grouped scatter mean with standard deviations between long saccades and short saccades in Healthy controls (HC), Parkinson's disease non-pareidolia type (PDnP) and Parkinson's disease pareidolia type (PDP). Red asterisks show significant univariate ANOVA differences after performing a multivariate ANOVA on both (A) frontal and (B) parietal group of electrodes. While 'Face' and 'Noise' categories were compared between all participant groups, 'Pareidolia' and 'Missed' were compared within Parkinson's disease patients only. For face, noise and pariedolia responses, parietal electrodes showed significant effect of saccade size but not for within groups. Similar effect was seen for pareidolia and noise responses in frontal electrodes. Of interest are pareidolic responses in PDP group which showed a significantly higher (positive) frontal presaccadic potential compared to PDnP group.

Supplementary Table 1 - 2 way ANOVA - Parametric and Non-parametric assessment for FRONTAL electrodes

**Response**	**Normality** **(Lilliefors corrected Kolmogorov-Smirnov test)**	**Equal Variance test**	**Histogram**	**Probability plot**	**Parametric ANOVA**		**Non-parametric ANOVA**	
					Main Effects		Main Effects	
					Saccade size *(Long, Short)*	Group *(HC, PDnP, PDP)*	Saccade size *(Long, Short)*	Group *(HC, PDnP, PDP)*
Face	$p < 0.05$	$p = 0.24$	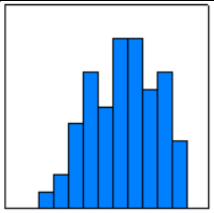	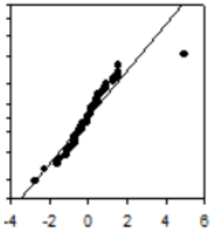	$F_{(1,55)} = 2.48$, $p = 0.121$	$F_{(1,55)} = 1.19$, $p = 0.310$	$F_{(1,55)} = 2.06$, $p = 0.156$	$F_{(1,55)} = 1.76$, $p = 0.181$
Noise	$p = 0.24$	$p = 0.51$	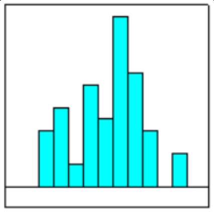	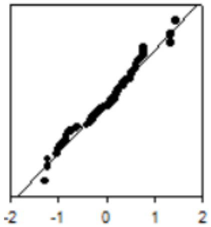	$F_{(1,56)} = 7.06$, ***$p = 0.010$***	$F_{(2,56)} = 1.72$, $p = 0.188$	$F_{(1,56)} = 8.83$, ***$p = 0.004$***	$F_{(2,56)} = 1.44$, $p = 0.246$
Pareidolia	$p < 0.05$	$p = 0.05$	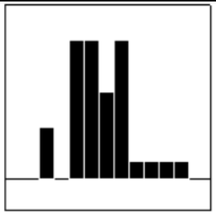	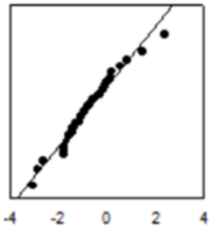	$F_{(1, 32)} = 2.19$, $p = 0.148$	$F_{(1, 32)} = 11.93$, ***$p = 0.002$***	$F_{(1, 32)} = 14.39$, ***$p < 0.001$***	$F_{(1, 32)} = 23.16$, ***$p < 0.001$***
Missed	$p = 0.06$	$p = 0.12$	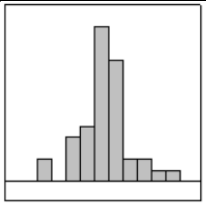	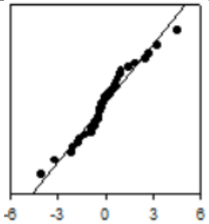	$F_{(1,38)} = 0.03$, $p = 0.855$	$F_{(1,38)} = 0.05$, $p = 0.815$	$F_{(1,38)} = 0.04$, $p = 0.848$	$F_{(1,38)} = 0.002$, $p = 0.961$

Supplementary Table 2 – 2 way ANOVA - Parametric and Non-parametric assessment for PARIETAL electrodes

**Response**	**Normality (Lilliefors corrected Kolmogorov-Smirnov test)**	**Equal Variance test**	**Histogram**	**Probability plot**	**Parametric ANOVA**		**Non-parametric ANOVA**	
					Main Effects		Main Effects	
					Saccade size *(Long, Short)*	Group *(HC, PDnP, PDP)*	Saccade size *(Long, Short)*	Group *(HC, PDnP, PDP)*
Face	$p < 0.05$	$p < 0.05$	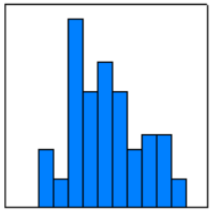	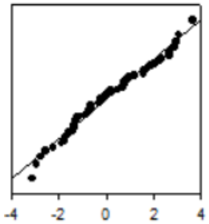	$F_{(1,55)} = 2.27$, $p = 0.138$	$F_{(1,55)} = 0.16$, $p = 0.848$	$F_{(1,55)} = 6.25$, $p = 0.150$	$F_{(1,55)} = 1.29$, $p = 0.283$
Noise	$p = 0.14$	$p = 0.84$	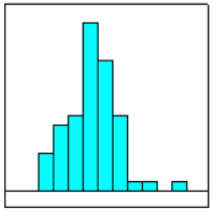	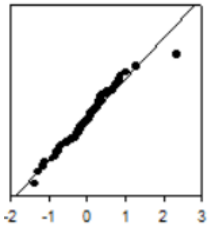	$F_{(1,56)} = 7.28$, ***p = 0.009***	$F_{(2,56)} = 4.34$, ***p = 0.018***	$F_{(1,56)} = 9.78$, ***p = 0.003***	$F_{(2,56)} = 3.47$, ***p = 0.038***
Pareidolia	$p < 0.05$	$p < 0.05$	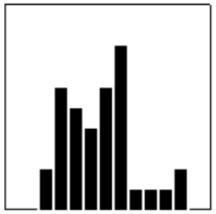	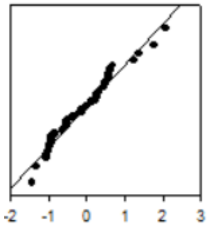	$F_{(1, 32)} = 8.77$, ***p = 0.006***	$F_{(1, 32)} = 0.86$, $p = 0.360$	$F_{(1, 32)} = 12.31$, ***p = 0.001***	$F_{(1, 32)} = 1.68$, $p = 0.204$
Missed	$p < 0.05$	$p = 0.65$	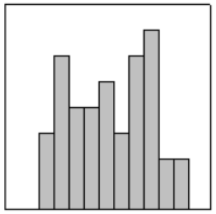	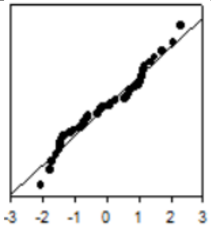	$F_{(1,38)} = 0.03$, $p = 0.855$	$F_{(1,38)} = 0.05$, $p = 0.815$	$F_{(1,38)} = 2.34$, $p = 0.134$	$F_{(1,38)} = 0.88$, $p = 0.354$

Supplementary Table 1 and Supplementary Table 2 – Shows results of 2-way ANOVA using parametric and non-parametric tests (when normality failed), Legend – HC - Healthy controls, PDnP - Parkinson's disease non-pareidolia type and PDP - Parkinson's disease pareidolia type. The normality statistic was set to 0.05 using Lilliefors corrected Kolmogorov-Smirnov test. For equal variance, the p-value was set to 0.05. If p-values were < 0.05, the test was considered to have failed normality or equal variances accordingly. Comparisons of Parametric and Non-parametric ANOVA for Main effects are shown in the subsequent columns. None of the response variables showed any significant interaction effects. Statistical significance was set to $p < 0.05$. Results used in the manuscript main text / table are shown in bigger font size.

References

Komogortsev OV, Gobert DV, Jayarathna S, Koh D-H, Gowda S. Standardization of automated analyses of oculomotor fixation and saccadic behaviors. IEEE Trans Biomed Eng 2010; 57

Nikolaev AR, Meghanathan RN, van Leeuwen C. Combining EEG and eye movement recording in free viewing: Pitfalls and possibilities. Brain Cogn 2016; 107: 55–83.

Olsen A, Matos R. Identifying parameter values for an I-VT fixation filter suitable for handling data sampled with various sampling frequencies. Eye Track Res Appl Symp ETRA 2012

Xue J, Quan C, Li C, Yue J, Zhang C. A crucial temporal accuracy test of combining EEG and Tobii eye tracker. Medicine (Baltimore) 2017; 96: e6444.
